# Cancer incidence estimates in adults aged 60 years and older living in low-and-middle-income countries for the years 2020 and 2040

**DOI:** 10.3332/ecancer.2023.1594

**Published:** 2023-08-31

**Authors:** Sophie Pilleron, Freddy Gnangnon, Vanita Noronha, Enrique Soto-Perez-de-Celis

**Affiliations:** 1 Department of Precision Health, Ageing, Cancer, and Disparities Research Unit, Luxembourg Institute of Health, 1A-B, Rue Thomas Edison, 1445 Strassen, Luxembourg; 2 Department of Visceral Surgery, National Teaching Hospital-Hubert Koutoukou Maga (CNHU-HKM), Avenue Pape Jean-Paul Il, 01 BP 386, Cotonou, Benin; 3 Department of Medical Oncology, Tata Memorial Hospital, Homi Bhabha National Institute, Mumbai 400012, India; 4 Department of Geriatrics, Instituto Nacional de Ciencias Medicas y Nutricion Salvador Zubiran, Mexico City 14080, Mexico; a https://orcid.org/0000-0001-7146-4740

**Keywords:** neoplasms, incidence, older adults, geriatric oncology, epidemiology, low-and-middle-income countries, developing countries, aged

## Abstract

Previous studies have shown a disproportionate rise in cancer incidence in low-and-middle-income countries (LMICs) due to rapid population ageing. This study aims to describe the cancer incidence in adults aged 60 years and older in LMICs to inform cancer control planning. Using the latest GLOBOCAN estimates for 2020, we describe the cancer incidence and the top five cancer sites among adults aged 60 years and older living in LMICs. We also project the incidence in 2040 by applying population projections, assuming no changes in incidence rates and risk profiles over time. In 2020, 6.3 million new cancer cases were diagnosed in older adults in LMICs, constituting over half of the global incidence burden (55%). In females aged 60 years and older living in LMICs, breast, lung, colon, stomach, and cervix uteri were the most frequent cancer types representing 51% of the total number of new cancer cases in older females. In males aged 60 years and older living in LMICs, lung, prostate, stomach, liver and colon were the most frequent cancer types representing 58% of the total number of new cancer cases in this subgroup. Variations were observed between income categories. The number of new cancer diagnoses in adults aged 60 years and older living in LMICs will almost double by 2040, reaching 11.5 million new cancer cases. The greatest increase is expected to happen in lower-income countries (+158% in lower-middle-income countries (excluding India) and +99% in low-income countries versus +38% in upper-middle-income countries). In conclusion, our findings call for an urgent adaptation of healthcare systems in LMICs by developing geriatric oncology and by including older adults in research, clinical guidelines, insurance schemes and cancer prevention policies.

## Background

Due to global population ageing, the absolute number of older adults with cancer is increasing worldwide [1, 2]. This increase is disproportionately affecting less developed regions. A previous study showed an expected +144% increase in the number of older adults with cancer in less developed regions versus +54% in more developed countries between 2012 and 2035 [1].

Despite the increasing older populations in low-and-middle-income countries (LMICs) and the existence of global strategies on ageing and health such as that of the World Health Organisation (WHO) [3], older adults are generally under-represented in research, health and clinical programmes, and those with cancer are often not included when defining global healthcare priorities [4].

Because of the heterogeneity of the older population in terms of health status and fitness, the lack of geriatric expertise in the oncology workforce and the scarcity of evidence-based cancer management strategies [5], the rising number of older people with cancer brings along challenges and opportunities for healthcare systems worldwide. Furthermore, the capability of healthcare systems to respond depends greatly on the availability of resources, including not only infrastructure but also personnel.

LMICs are a heterogeneous group of countries between and within the World Bank income categories (low, lower-middle and upper-middle) in terms of demographic, healthcare system, infrastructures, medical and paramedical expertise, culture and traditions.

There is an urgent need to describe the cancer burden among the older population living in LMICs and to discuss their specific challenges to improve the allocation of resources to control the cancer burden in developing regions of the world.

Using 2020 GLOBOCAN estimates, we described cancer incidence estimates in adults aged 60 years and older living in LMICs. We also projected the number of new cancer cases expected to be diagnosed in adults aged 60 years and older living in LMICs in 2040.

## Methods

Older adults were defined as adults aged 60 years and older, following the United Nations (UN) definition, which takes into account the characteristics of ageing in developing regions of the world [6].

LMICs were selected using the 2020 World Bank classification [7].

The estimated number of new cancer cases, as well as the population size for the year 2020 for the world and 185 countries, was extracted from the Global Cancer Observatory [8]. Data were available for 34 cancer types and all cancer sites combined, excluding non-melanoma skin cancer (ICD-10 C00-97, except C44), and for ten age groups (0–14, 15–39, 40–44, 45–49,50–54, 55–59, 60–64, 65–69, 70–74, 75 and over) by sex.

Numbers of new cases among adults aged 60 years and older and truncated age-standardised incidence rates for the 60-year-and-older age group (ASR per 100,000 inhabitants of the same age – world standard population of Segi, revised by Doll* et al* [9]) for all cancer sites combined and the five most common cancer sites were described for low-income, lower-middle and upper-middle World Bank income separately. Given their relative population sizes, estimates for China (upper-middle-income country) and India (lower-middle-income country) were presented separately.

We also computed the number of new cancer cases occurring in adults aged 60 years and older as a proportion of the total cancer diagnoses in both sexes and by sex, worldwide, and by the World Bank income group.

Finally, we predicted the future number of new cancer cases (all cancer sites combined) in adults aged 60 years and older worldwide and by World Bank income group for the year 2040 by applying sex-and age-specific incidence rates in 2020 to UN population projections using the medium-fertility variant for the year 2040 [10].

For our projections, we considered only the effect of ageing and population growth and assumed no change in the risk patterns of cancer incidence between 2020 and 2040.

Data analysis was conducted using R statistical software (version 4.2.2; R Development Core Team, 2022) [11].

## Results

### Overall cancer burden

In 2020, an estimated 6.3 million new cancer cases were diagnosed among adults aged 60 years and older living in LMICs (55.3% of cases in adults aged 60 and older worldwide), representing 55.6% of all new cancer cases (all ages combined) in LMICs (Table 1). This proportion increased as World Bank income increased, going from 39.2% in low-income countries to 59.3% in upper-middle-income countries (excluding China), with the proportion in lower-middle-income countries (excluding India) being in-between at 47.4%. The proportion of cancer cases diagnosed in adults aged 60 years and older in China (which alone accounted for 59% of all cases diagnosed in upper-middle-income countries) and India (43% of all cases diagnosed in lower-middle-income countries) were 60.0% and 47.8%, respectively.

At the country level, Bulgaria, an upper-middle-income country, had the highest proportion of total cancers diagnosed in adults aged 60 years and older (73%), and Malawi, a low-income country, had the lowest (23% – Supplemental Table 1 at https://doi.org/10.6084/m9.figshare.22561162.v1).

**Figure 1.**
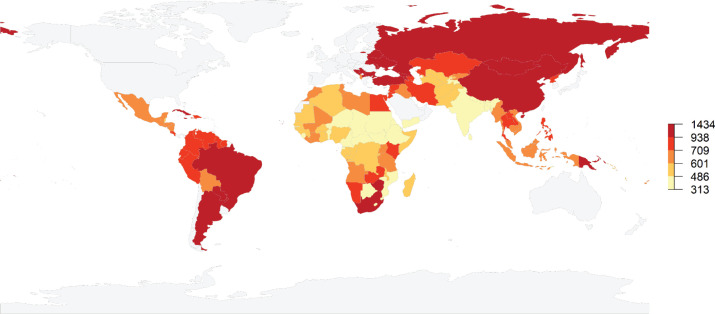
Map showing quantiles of estimated truncated age-standardised incidence rates for all types of cancers (excluding non-melanoma skin cancer – per 100,000 inhabitants) among adults aged 60 years and older living in LMICs in 2020. Estimates are shown for LMICs only. Grey colour indicates no value.

Truncated age-standardised incidence rates (ASR) in LMICs were 821 per 100,000 but varied greatly, going from 540 in lower-middle-income countries to 1,005 per 100,000 inhabitants aged 60 years and older in upper-middle-income countries (Figure 1). There were also great variations within World Bank income groups. In low-income countries, truncated ASRs varied between 312 per 100,000 in Niger to 834 per 100,000 in the Democratic People’s Republic of Korea (Supplemental Table 1 at https://doi.org/10.6084/m9.figshare.22561162.v1). Truncated ASRs in lower-middle-income countries ranged from 374 in Djibouti to 1,243 in Mongolia, while in upper-middle-income countries, they ranged from 410 in Equatorial Guinea to 1,434 in Serbia.

### Cancer profile in older adults living in LMICs by sex

**Figure 2.**
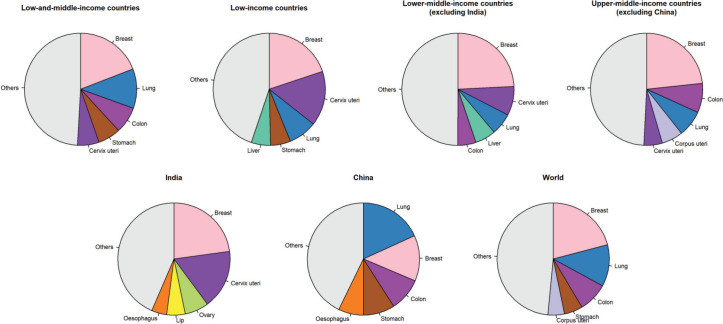
Pie charts representing the five most common cancer sites in females aged 60 years and older by World Bank income categories.

In females aged 60 years and older living in LMICs, breast, lung, colon, stomach, and cervix uteri were the most frequent cancer types and represented 50.8% of the total number of new cancer cases in this age group (Figure 2). The top five cancers were breast, cervix uteri, lung, stomach and liver cancers (55% of all cancers diagnosed in this age group) in low-income countries. In lower-middle-income countries (excluding India), the top three were the same as those in low-income countries, followed by liver and colon cancers (50%). The cancer profile was different in India with the five most common cancer types in females aged 60 years and older being breast, cervix uteri, ovarian, lip and oral cavity, and oesophageal cancers (56%). In upper-middle-income countries, breast, colon, lung, corpus uteri, and cervix uteri were the five most common cancer types (51%). In China, lung cancer was the most common cancer diagnosed in females aged 60 years and older, followed by breast, colon, stomach, and oesophageal cancers; with these cancers representing 57% of all cancers diagnosed in this age group. As a comparison, the most common cancer types worldwide in females aged 60 years and older were breast, lung, colon, stomach and corpus uteri (52%).

**Figure 3.**
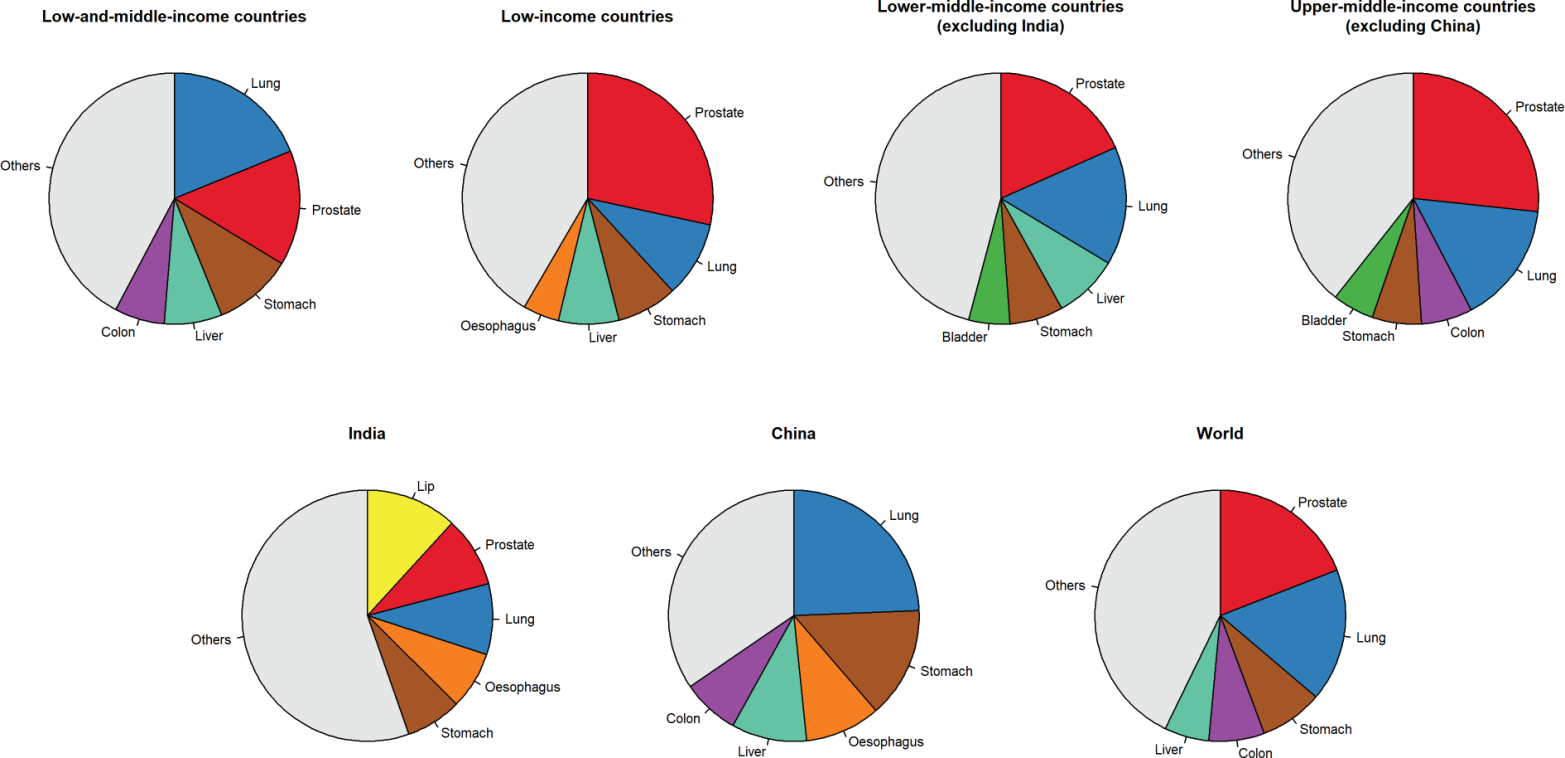
Pie charts representing the five most common cancer sites in males aged 60 years and older by World Bank income categories.

In males aged 60 years and older living in LMICs, lung, prostate, stomach, liver, and colon were the most frequent cancer types and represented 58% of the total number of new cancer cases in this age group (Figure 3). Like females, some differences were observed between World Bank income groups. In low-and lower-middle-income countries (excluding India), prostate, lung and stomach cancers were the three most cancer types, followed by liver and oesophageal cancers in low-income countries and stomach and bladder in lower-middle-income countries. The top five cancers represented 58% and 54% of all cancer diagnosed in males aged 60 years and older, respectively. In India, cancers of the lip and oral cavity were the most frequently diagnosed in older males, followed by prostate, lung, oesophagus and stomach (45%). In upper-middle-income countries (excluding China), prostate, lung, colon, stomach and bladder cancers were the most frequent cancers, representing 61% of all cancers diagnosed in this population. Like females, lung cancer was the most common cancer type diagnosed in older males in China, followed by stomach, oesophagus, liver, and colon cancers. These top five sites accounted for 72% of all cancers diagnosed in older males in China. Worldwide, the top five cancers in males aged 60 years and older were prostate, lung, stomach, colon and liver, representing 57% of all cancer cases diagnosed in this age group.

### Projected number of new cancer cases in older adults living in LMICs by 2040 (Figure 4)

An estimated 11.5 million new cancer cases (66% of all cases, all ages combined) are expected to be diagnosed in adults aged 60 years and older living in LMICs by 2040, representing 57% of the worldwide expected number of cases in this age group.

Compared to the 2020 figure, this represents an expected increase of +84% in the number of new cancer cases aged 60 years and older living in LMICs (compared to an increase of +78% worldwide). The most significant increase is expected in lower-middle-income countries (excluding India) which can expect to see their number of cases in older adults increase by +158% (or an additional 1.3 million new cancer cases) in the next 20 years. Although this increase is expected to be more moderate in low-income countries, the number of new cancer cases is still expected to double by 2040 (+99%). In upper-middle-income countries (excluding China), the expected increase would be +38%. China and India will see the number of cancers diagnosed in adults aged 60 years and older double by 2040, with increases of +96% and +95%, respectively, or an additional 2.6 and 0.6 million new cancer cases aged over 60, respectively.

## Discussion

Using the most up-to-date estimates from the global cancer observatory, we showed over 6 million new cancer cases (excluding non-melanoma skin cancer) were diagnosed in adults aged 60 years and older living in LMICs in 2020. This number is projected to reach 11.5 million in 2040 if the cancer risk pattern does not change over the period. The greatest increase is expected to occur in lower-middle-income countries (excluding India) which will see an increase of +158% in the number of cancer diagnoses among adults aged 60 years and older. Breast cancer in older females and lung and prostate cancers in older males were, by far, the most common cancer types diagnosed in 2020 regardless of the World Bank income category, except for China. The considerable increase in the number of cancer diagnoses among older adults in LMICs will pose a substantial strain on healthcare systems which will need to adapt quickly to offer optimum comprehensive care for ageing populations.

Infection-related cancers, mainly cervical cancers in older females, and gastric and liver cancers in both sexes were important contributors to the cancer burden in low-income and lower-middle-income countries. The importance of the contribution of colon cancer which is mainly caused by unhealthy dietary and physical activity habits is greater in upper-middle-income countries compared to other World Bank income categories. These differences in cancer patterns mean that preventive strategies need to be tailored to the local cancer profile. For example, strategies to reduce the likelihood of infection over the lifetime such as vaccination for hepatitis B, responsible for liver cancer, and Human Papilloma Virus, responsible for cervical cancers (as well as other malignancies), while benefitting future generations of older adults worldwide, would more significantly benefit those living in LMICs, where these cancer types are more common. In contrast, preventive interventions targeting smoking, obesity, physical inactivity and unhealthy diet throughout life, even at older ages [12–15], would have a greater impact in wealthier countries, although such interventions will be relevant for less-resourced countries as well.

As previously described for adults aged 65 years old [1], lung cancer is the most common cancer site diagnosed in both females and males aged 60 years and older in China. The high prevalence of smoking is likely to explain this finding in Chinese males but not in Chinese females, who have low smoking rates [16]. Lung cancer in Chinese females may, however, be caused by exposure to second-hand smoke, household air pollution originating from heating stoves using biomass, coal and other solid fuels, or outdoor air pollution particulate matter [17]. The State Council of China enacted the ‘Air Pollution Prevention and Control Action Plan’ in 2013 to lower the particulate matter level and a recent study showed an encouraging reduction in chronic conditions of the respiratory and circulatory systems [18]. This may also reduce the risk of lung cancer in future cohorts of older adults. However, this could be counterbalanced by an increasing trend in smoking in younger generations of Chinese males [16].

Many LMICs face a lack of infrastructure, access to anti-cancer treatment, oncologists and radiotherapists to offer optimal cancer management, regardless of the age of the patient. In addition, cancer management in older adults requires geriatric expertise, which is crucially lacking across many LMICs [19]. While some initiatives, such as those from the International Society for Geriatric Oncology (SIOG) offer geriatric oncology training in some LMICs [20], there is a huge need to be innovative in delivering care to older adults with cancer in low-resource settings. Such interventions could include the involvement of nurses or community health workers who can be trained in performing a geriatric assessment and implementing evidence-based interventions. Many models of care already exist for providing care that includes geriatric principles for older adults with cancer, and randomised clinical trials have shown that such interventions may lead to improved outcomes [21]. However, those studies have been performed in high-income countries, and research on how best to implement geriatric oncology in LMIC settings, including the use of innovative technologies such as telemedicine or mobile health, is urgently needed. Such research would certainly require adequate funding and global collaboration, and as such, this represents one of the highest priorities for the global development of geriatric oncology according to SIOG [22].

Offering palliative and supportive care for patients and their families represents a human right and is becoming increasingly important in high-income countries. Still, it remains a low priority and an under-researched area in LMICs, especially in older adults [23]. This represents a huge gap in cancer care since it is foreseeable that a significant proportion of older patients diagnosed with cancer in LMICs will present with advanced disease, where preserving quality of life, alleviating pain, and controlling symptoms is the most important and relevant goal. The Multinational Association of Supportive Care in Cancer defines supportive care as ‘*the prevention and management of the adverse effects of cancer and its treatment. This includes management of physical and psychological symptoms and side effects across the continuum of the cancer journey from diagnosis through treatment to post-treatment care’* [24]. Resource-stratified guidelines as those proposed by the Breast Health Global Initiative, but tailored to the unique needs of older adults and their caregivers would help offer optimal supportive care for the increasing ageing population living in LMICs [23].

Based on the Global Atlas of Palliative Care, only half of the countries in the world, regardless of the World Bank income category, have an operational non-communicable disease plan including palliative care [25]. While 91% of high-income countries have dedicated funding for palliative care within their healthcare systems, this only happens in 48% of low-income countries [25]. When it comes to effective access to palliative care, 27% of patients living in upper-middle-income countries had access to palliative care, but only 3% and 1%, respectively in lower-middle-income and low-income countries (against 69% in high-income countries) [25].

The forecasted increase in cancer incidence among older adults in LMICs will also pose a considerable financial burden on households. Older adults in many LMICs support their families and are still active, mainly doing informal or unpaid jobs [26]. In addition, the costs of cancer diagnosis, care, and curative or palliative treatment are high and universal health coverage is not a reality for many LMICs. Between 18% and 93% of patients with cancer in LMICs face objective financial toxicity [27], and older patients with cancer have a higher likelihood of catastrophic health expenditure [28]. Implementing a health financing system to reduce (or avoid) out-of-pocket payments should be a priority in every country.

Our study has limitations. The definition of older adults differs between disciplines (e.g., medicine versus economics) and settings due to different life experiences and risk profiles. For instance, although the age of 50–55 was suggested to ‘define’ an older adult living in Africa, where life expectancy is lower [29], this would not be appropriate for super-aged societies such as Japan. However, for the purpose of description, we chose to use the UN definition of 60 years and older. Although we acknowledge this age cut-off may not be relevant in some settings, we believe it is the one that most correctly captures the current panorama of ageing in developing regions.

As described in Ferlay *et al* [30], many LMICs have no cancer registry and Globocan estimates are based on incidence data from neighbouring countries or from cause-specific mortality data. Mortality data relies on the accuracy of the cause of death reported on the death certificate when they exist. Identifying the cause of death may be challenging in older adults because of other fatal conditions, for instance. There is a risk of under-ascertainment of cancer cases in older adults which would mean that rates and the number of cases would be underestimated.

Finally, our projections of the number of cancer cases in older adults in LMICs in 2040 did not consider trends in cancer incidence and changes in risk factors. However, the possible decrease in infection-related cancers in LMICs in the next decades will likely be compensated by an increase in the incidence of lifestyle-related cancers, especially in countries in epidemiological transition [31, 32].

## Conclusion

Most older adults with cancer already live in LMICs, and their number is still rising, especially in less-resourced countries, and this will greatly increase during the next 20 years. To close the cancer care gap and improve outcomes, it is urgent to include older adults with cancer in national, regional, and global cancer control policies. This should ideally lead to the development of age-friendly healthcare systems, with geriatric expertise embedded into each and every aspect of care as recommended by the WHO. To achieve this, researchers, clinicians and policymakers should take into account SIOG recommendations and foster the development of geriatric oncology resources that are tailored to each country’s needs, priorities, and resources [22].

## List of abbreviations

ASR: age-standardised rates; LMIC: Low-and-middle-income countries; SIOG: International Society for Geriatric Oncology; UN: United Nations; WHO: World Health Organisation.

## Conflicts of interest

The author(s) declare that they have no conflict of interest.

## Funding

This work was not externally funded.

## Author contribution

SP: Conceptualisation; formal analysis; writing – Original draft; writing – review and editing; visualisation.

FG, VR, ES: Writing – Review and editing.

## Data availability

Incidence data and 2020 population size are openly accessible at https://gco.iarc.fr/ and the population size projections for the year 2040 are available at https://population.un.org/wpp/.

## Ethical considerations

All data used in this work are publicly available; ethics approval was not required.

## Figures and Tables

**Figure 4.**
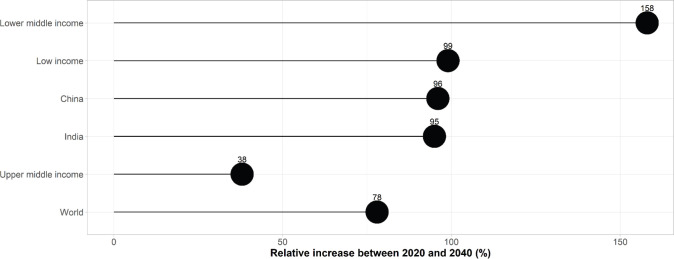
Estimated relative increase in the number of new cancer cases among adults aged 60 years and older living in LMICs between 2020 and 2040 (%).

**Table 1.** Number of new cancer cases, percentages of new cancer cases and adults aged 60 years and older, crude and truncated ASR among adults aged 60 years and older living in LMICs and worldwide by sex.

	Number of new cases among 60+^a^		Percentages of new cases among 60+ (%)		Percentages of population aged 60+ (%)		Crude incidence rate among adults aged 60+ (per 100,000)		Truncated age-standardised incidence rates among adults aged 60+ (per 100,000)	
	Female	Male	Female	Male	Female	Male	Female	Male	Female	Male
LMICs	2,748,000	3,509,000	49.3	61.7	12.3	10.4	689	1,015	668	1,007
Low-income countries	99,000	95,000	34.6	47.6	5.7	4.6	505	597	500	608
Lower-middle-income countries (excluding India)	378,000	427,000	42.4	52.9	8.5	7.1	562	763	553	763
India	292,000	336,000	43.3	52.6	10.7	9.6	411	491	407	491
Upper-middle-income countries (excluding China)	860,000	1,043,000	52.6	66.3	14.7	11.7	782	1,230	748	1,205
China	1,120,000	1,607,000	53.8	65.2	18.6	16.3	853	1,332	823	1,317
World	4,925,000	6,381,000	56.3	68.3	14.6	12.3	871	1,318	816	1,268

a Rounded to the nearest thousand
